# Human pluripotent stem cell differentiation to functional pancreatic cells for diabetes therapies: Innovations, challenges and future directions

**DOI:** 10.1186/s13036-017-0066-3

**Published:** 2017-07-03

**Authors:** Elena F. Jacobson, Emmanuel S. Tzanakakis

**Affiliations:** 1 Department of Chemical and Biological Engineering, Tufts University, 4 Colby St., Room 276A, Medford, MA 02155 USA; 2 Tufts Clinical and Translational Science Institute, Tufts Medical Center, Boston, MA 02111 USA

**Keywords:** Pluripotent stem cells, Embryonic stem cells, Induced pluripotent stem cells, Pancreatic differentiation, Endocrine cells, Exocrine cells, Pancreas, Pancreatic β-cells, Diabetes

## Abstract

Recent advances in the expansion and directed pancreatogenic differentiation of human pluripotent stem cells (hPSCs) have intensified efforts to generate functional pancreatic islet cells, especially insulin-secreting β-cells, for cell therapies against diabetes. However, the consistent generation of glucose-responsive insulin-releasing cells remains challenging. In this article, we first present basic concepts of pancreatic organogenesis, which frequently serves as a basis for engineering differentiation regimens. Next, past and current efforts are critically discussed for the conversion of hPSCs along pancreatic cell lineages, including endocrine β-cells and α-cells, as well as exocrine cells with emphasis placed on the later stages of commitment. Finally, major challenges and future directions are examined, such as the identification of factors for in vivo maturation, large-scale culture and post processing systems, cell loss during differentiation, culture economics, efficiency, and efficacy and exosomes and miRNAs in pancreatic differentiation.

**Electronic supplementary material:**

The online version of this article (doi:10.1186/s13036-017-0066-3) contains supplementary material, which is available to authorized users.

## Background

Diabetes mellitus refers to a group of chronic metabolic disorders characterized by hyperglycemia due to autoimmune destruction of insulin-producing beta(β)-cells (type 1 diabetes; T1D) or to extensive β-cell exhaustion and depletion often exacerbated by insulin resistance (type 2 diabetes; T2D). Almost 9% of the population in the US and worldwide (ages 20–79) have diabetes and diabetes-associated healthcare costs are among the highest for major maladies [1, 2]. All T1D and many T2D patients require insulin administration to control their blood glucose. Insulin injections result in suboptimal glucose control because of the multifactorial nature of glucose homeostasis, which is affected by diet, physical activity and metabolic patterns. This makes proper hormone dosage and its timing challenging [3]. Whole pancreas or pancreatic islet (~6–10 × 10^5^ islets or ~10^9^ β-cells [4, 5]) transplantation has the potential to restore normoglycemia [6] without the use of exogenous insulin. This approach, however, is severely limited by the shortage of donor tissue and the requirement for immunosuppression to prevent rejection of the transplanted islets.

Human pluripotent stem cells (hPSCs; including embryonic (hESCs) and induced pluripotent stem cells (hiPSCs)) can serve as a renewable source of differentiated cells and tissues due to their capacity for extensive expansion and commitment to various somatic cell fates. In particular, recent advances in directing hPSCs to pancreatic cell phenotypes, especially insulin-producing β-cells, have sparked hope for cell therapies for diabetes [7–9]. Various other cell types, including progenitor cells (e.g., mesenchymal stem cells (MSCs) and umbilical cord cells [10, 11]) and fibroblasts [12], have been reported to yield islet cells. However, this review will be limited to studies using hPSCs as starting materials for producing pancreatic cells.

Challenges in coaxing hPSCs to mature functional β-cells that mimic the properties of native islet cells have hampered progress over the past decade. We will review the basic concepts of pancreatic organogenesis, which has served as the basis for designing differentiation strategies. Then we will highlight and discuss recent studies demonstrating the pancreatogenic specification of hPSCs. Finally, we will examine challenges and future directions in the field like identification of factors for in vivo maturation, large-scale culture and post processing systems, cell loss during differentiation, culture economics, efficiency, and efficacy and exosomes and miRNAs in pancreatic differentiation.

### Pancreas development

**Fig. 1:**
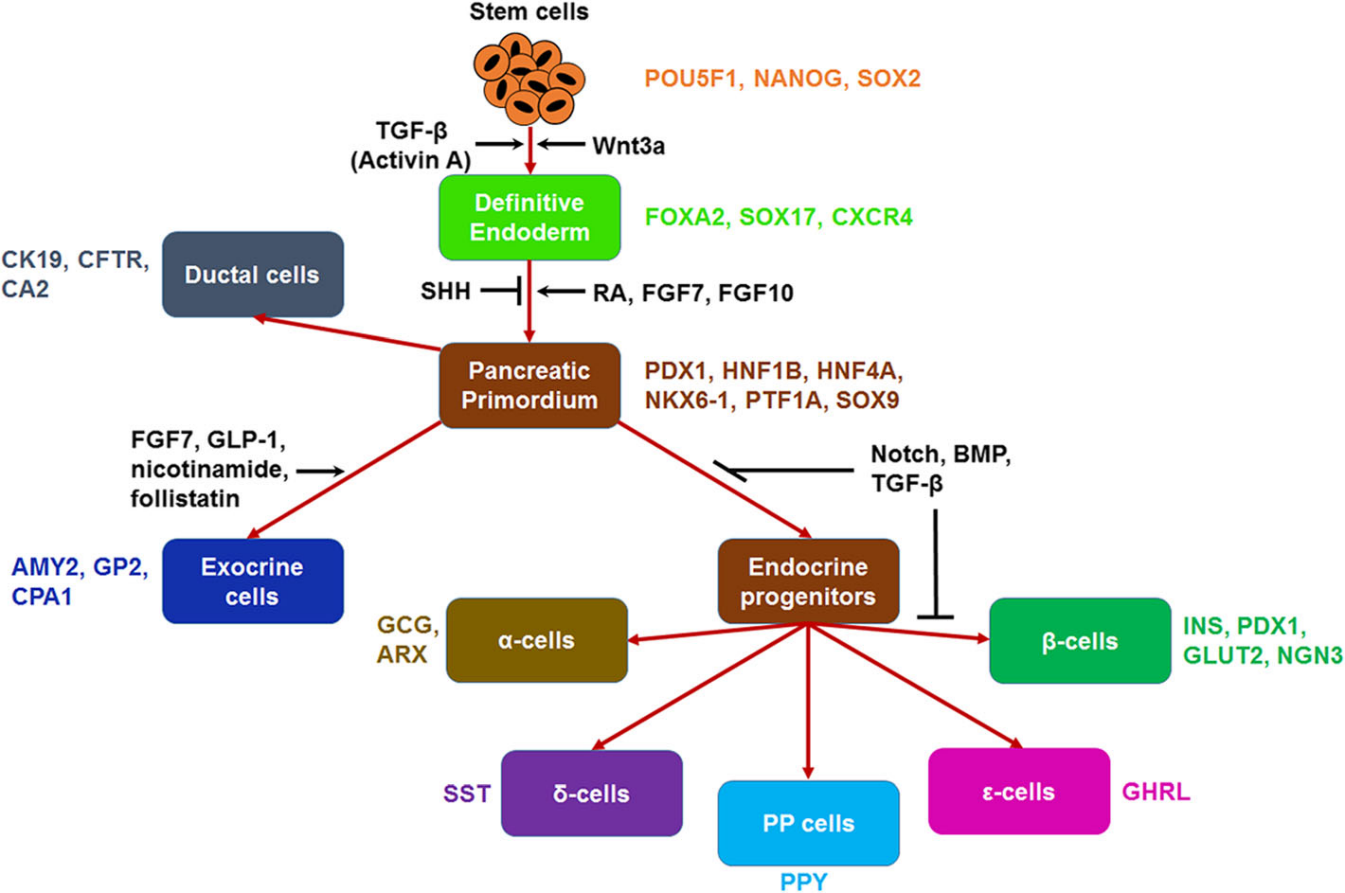
Pancreatic differentiation of stem cells: A schematic is shown depicting various lineages of pluripotent stem cell differentiation toward pancreatic cell progeny. Differentiation cues and signaling pathways are shown (*black fonts*) as well as markers (*colored fonts*) for various cell types. SHH: sonic hedgehog, RA: retinoic acid, FGF7: fibroblast growth factor-7 (also known as keratinocyte growth factor (KGF)), FGF10: fibroblast growth factor-10, GLP-1: glucagon-like peptide-1, BMP: bone morphogenetic protein

There are several excellent reviews on pancreas development informed primarily by studies on animal models [13, 14] rather than on human embryonic pancreatic tissue [15]. Here, we provide a simplified description of the formation of pancreas in vivo with emphasis on major instructive signals directing cell specification. Protocols for the conversion of hPSCs to pancreatic cells in vitro recapitulate aspects of these signaling events (Fig. 1). Definitive endoderm (DE) and mesoderm arise from the epiblast during gastrulation. The DE then folds to form the primitive gut tube segmented into foregut, midgut and hindgut in an anterior-posterior orientation, surrounded by mesenchyme and the notochord. While the midgut and hindgut regions give rise to the small intestine and colon, the foregut is the source of cells for multiple tissues and organs of the respiratory and digestive tract, such as the lung, esophagus, thyroid, stomach, liver and pancreas. Signals, including fibroblast growth factor (FGF) 4 and retinoic acid (RA), mediate the commitment of foregut endoderm to pancreatic anlage [16, 17].

Pancreatic development at this point occurs at two distinct areas, the ventral and dorsal anlagen, which later fuse into a single organ. Despite differences in the specification programs of these areas, the notochord secretes activin and FGF2, which repress sonic hedgehog (Shh) signaling in the patterning of the pancreatic endoderm as shown in chick embryos, leading to the appearance of PDX1 positive (PDX1^+^) epithelium [18, 19]. Expansion of these precursor cells is mediated by mesenchymal factors. The epidermal growth factor (EGF) stimulates the proliferation of early pancreatic progenitor cells but inhibits endocrine differentiation. Similarly, FGF ligands targeting the FGF receptor 2B (FGF1, FGF7 and FGF10) most likely have a role consistent with that of FGF10 in mice, acting as mitogens of progenitor cells in the developing pancreas [20]. WNT signaling also appears to promote the proliferation of human pancreatic progenitors [21, 22] similar to its role in mice [23]. The roles of Notch, RA and bone morphogenetic protein (BMP) signaling during human pancreas development are less well understood. In particular, Notch signaling is activated by the mesenchymal FGF10 and influences the expansion of undifferentiated pancreatic progenitor cells expressing *Pdx1*, *Nkx6–1* and p48/*Ptf1a* as experiments in mice have shown [24]. These cells give rise to the endocrine and exocrine compartments of the pancreas.

The acinar and ductal cells comprising the exocrine tissue are specified by Wnt-activating ligands and mesenchymal release of FGF10, FGF7, laminin-1 and follistatin, in addition to Notch signals. Acinar differentiation is regulated by a set of transcription factors including Ptf1a and Mist1 [25]. Ptf1a forms a complex with Tcf12 and Rbpjl, which allows the expression of genes for the secretory enzymes present in the mature acini [26]. Acinar cells secrete digestive enzymes such as trypsin, chymotrypsin, lipase, amylase and carboxypeptidase A1 (CPA1) [27]. Ductal cell-specific transcription factors are not as well-known but HNF1B and HNF6 are thought to be active in this cell type. Ductal cells form tubular networks, secrete bicarbonate and mucins and are ciliated and polarized [26]. They express cytokeratin-19 (KRT19), cystic fibrosis transmembrane receptor (CFTR), carbonic anhydrase II (CA2) and *Dolichos biflorus* agglutinin (DBA) lectin.

Expression of the transcription factor neurogenin 3 (NGN3) increases concomitantly with the emergence of human fetal β-cells whereas SOX9 is absent in endocrine cells [28] (but not in acinar cells). The expression of NGN3 in human fetuses is transient and peaks toward the end of the first trimester and becomes undetectable after week 35 [29]. Other transcription factors, such as PDX1, NKX6–1, PAX6, NEUROD1 and NKX2–2, are also displayed by endocrine cells starting at 8 weeks post-conception [15]. It should be noted that NKX2–2 is not detected before endocrine progeny becomes apparent [28] in contrast to its broad expression in the murine pancreatic bud until E13, when it becomes restricted to NGN3-expressing progenitor cells [30]. Epithelial progenitor cells migrate into the mesenchyme and form islets consisting of alpha (α), β, delta (δ), pancreatic polypeptide (PP) and epsilon (ε) cells, which produce glucagon (*GCG*), insulin (*INS*), somatostatin (*SST*), PP (*PPY*) and ghrelin (*GHRL*), respectively (genes encoding the corresponding hormones are noted).

Much of what is known about pancreatic development is derived from animal studies. As many studies have shown however, there are important differences between pancreatic development in animals and humans. Thus, better understanding of human pancreas formation in vivo will be essential for developing efficient methods of in vitro pancreatogenic differentiation of hPSCs to functional islet cells.

### In vitro differentiation of hPSCs to pancreatic endocrine cell progeny

The production of insulin-producing cells resembling native β-cells from hPSCs has been the primary focus of research in the field. However, this does not lessen the importance of the other endocrine cell types (principally α-cells), which function in an integrated manner with β-cells within native islets to maintain blood glucose homeostasis. Therefore, the generation of β-cells is reviewed first, followed by reports on the generation of glucagon-producing α-cells.

### Derivation of β-cell-like cells

**Fig. 2:**
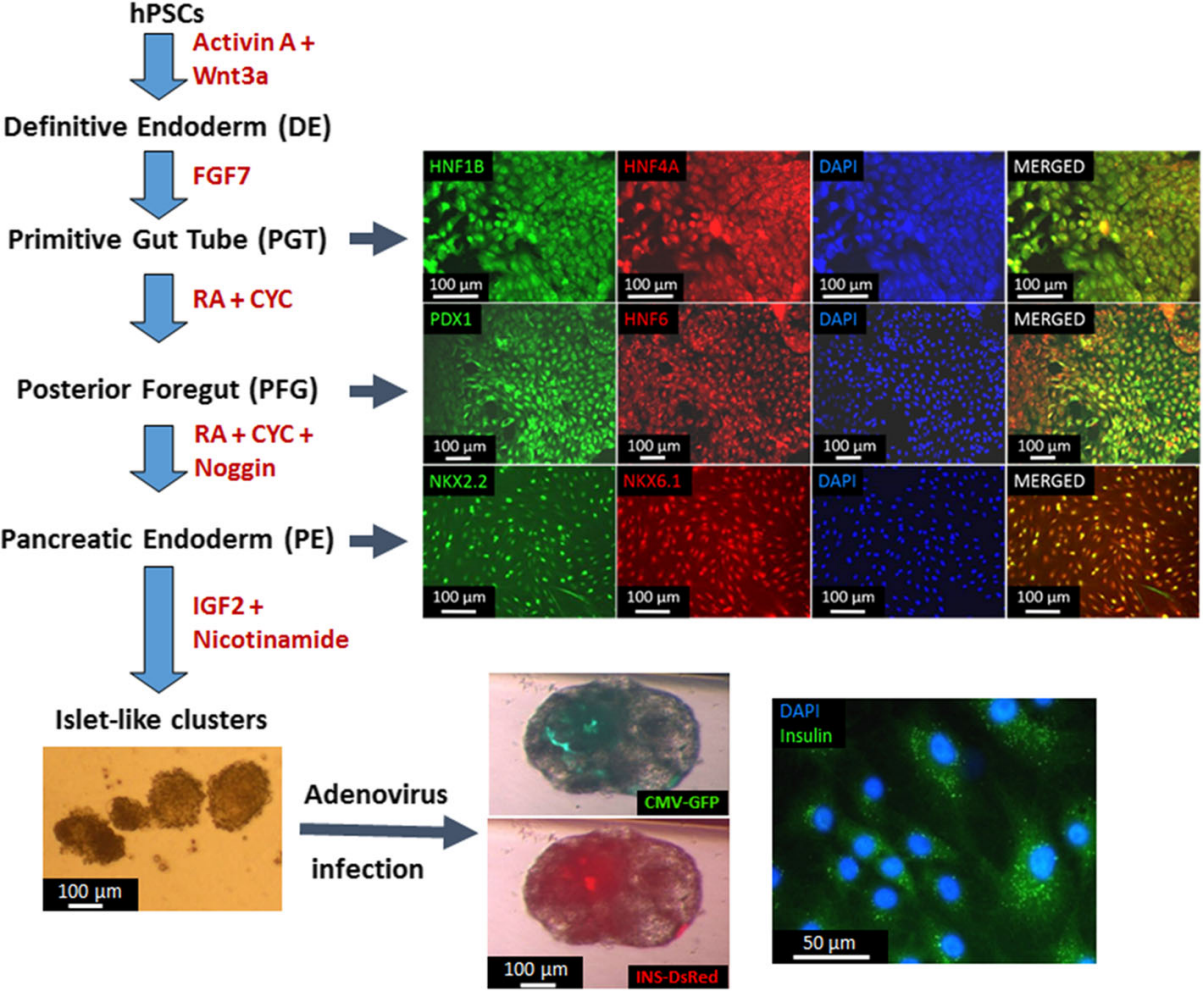
Pancreatic differentiation [36] of hPSCs (H9 hESCs) modified from Kroon et al. [41]. The factors used for differentiation are shown in red font (CYC: KAAD-cyclopamine, IGF2: insulin-like growth factor 2). Islet-like clusters were infected with a recombinant adenovirus carrying a bicistronic cassette with the green fluorescent protein (GFP) under the cytomegalovirus (CMV) promoter and the DsRed reporter under the rat insulin promoter. Immunostainings for specific markers at each stage are shown

Early work on pancreatogenic differentiation was performed with mouse ESCs (mESCs) and relied on the use of agents such as serum, dimethyl sulfoxide (DMSO) and nicotinamide, which have pleiotropic effects [31–33]. These methods were also applied to subsequent work with hESCs [34], but the low efficiency of differentiation and the challenges in the characterization of the cell progeny prompted researchers to consider methods mimicking aspects of pancreas development. Using a 5-step process, D’Amour et al. [35] differentiated hESCs in succession to cells expressing markers of DE, primitive gut tube, posterior foregut, pancreatic endoderm and endocrine precursors and finally to hormone-expressing endocrine cells (Additional file 1: Table S1). While some of the resulting cells expressed insulin, most of the cells were multihormonal co-expressing combinations of INS, GCG and SST. An average of 7% of the cells were INS^+^ and 13% were SYP^+^ (synaptophysin) at day 16 of differentiation as assessed by flow cytometry. Compared to primary adult human islets, the final population of differentiated hPSCs displayed substantially higher levels of proinsulin. A 2- to 6-fold increase in C-peptide was seen in response to KCl and a 30–300% increase in C-peptide release was measured over basal levels in 10% of the cells when exposed to glucose. The insulin content of the INS^+^ cells ranged from 14 to 208 pmol/μg DNA, which is comparable to that of primary adult human islets (58–310 pmol/μg DNA). Treatment with tolbutamide led to a 3- to 7-fold increase in C-peptide release, suggesting the existence of functional K^+^_ATP_ channels in these cells. Electron micrographs revealed the presence of secretory granules with a mixture of morphologies including some with clear halos surrounding dense cores, which is the phenotype of human β-cells insulin secretory granules [35]. A modified version of this protocol was developed in our laboratory for hPSC differentiation toward islet cells (Fig. 2 **)** [36]**.**

Despite the vast improvements in producing pancreatic progeny from hPSCs with this protocol, the C-peptide released in response to glucose was minimal, the quantity of proinsulin in the cells was too high, many of the cells were polyhormonal and the overall differentiation efficiency was low. The marginal C-peptide release in response to glucose and the high content of proinsulin suggests problems with the cells’ glucose sensing, processing and insulin secretion circuitries. Of note, the fetal pancreas responds to tolbutamide but not glucose [37]. Moreover, polyhormonal cells (e.g. GCG^+^/INS^+^) are detected in human fetal pancreas but their numbers are reduced from the start of endocrine differentiation and are almost undetectable in the adult pancreas [38, 39]. These findings prompted the authors to hypothesize that the differentiated hPSCs resemble immature fetal endocrine cells. The nature and potential of these multihormonal cells remain unclear, but direct lineage tracing in the mouse showed that mature α- and β-cells result from independent lineages that never co-express INS and GCG [40]**.**

From a practical standpoint, the low efficiency (~7% INS^+^ cells) and the use of a single hESC line (CyT203, Table 1) indicate that additional differentiation factors and cell line-specific optimization would be necessary. The same group published a subsequent study in which pancreatic endoderm and endocrine precursors derived from hESCs were implanted into the epididymal fat pads of severe combined immunodeficient (SCID) mice. This effectively promoted cell maturation in vivo [41]. Very low serum levels of human C-peptide were detected in response to glucose administration as early as day 30 post-implantation and after 3 months. In addition, fasting glucose-stimulated serum levels of human C-peptide were similar to those in mice implanted with 3-5 × 10^3^ adult human pancreatic islets. Explanted hESC-derived cells post in vivo maturation exhibited a mature phenotype with independent expression of INS, GCG, SST, PPY and GHRL and the transcription factor MAFA was expressed in INS^+^ but not GCG^+^ cells. There was also co-localization of the prohormone-processing enzyme, proprotein convertase subtilisin/kexin type 1 (PCSK1), in INS^+^ but not GCG^+^ cells and the expression of PCSK2 in both cell types. Furthermore, the hESC-derived endocrine cells rescued mice from streptozotocin (STZ)-induced diabetes. The experiments however, were conducted with two hESC lines (CyT49 and CyT203) with notable differences among the committed progeny, indicating that optimization would be required for each hPSC line.

**Table 1:** Cell lines used for in vitro differentiation

		Reference
hESC line/s	HUES8	[8, 53]
	H1	[9, 53]
	hES-3	[58]
	MEL1	[59]
	CyT203^a^, CyT25, CyT49, BG01, BG02, BG03	[35]
hiPSC line/s	hiPSC-1, hiPSC-2	[8]
	Gibco Human Episomal iPSC	[53]

^a^System optimized for these cells

In a recent report [8], the Melton group described the generation of glucose-responsive cells from hPSCs following a similar approach of coaxing cells through different stages mimicking pancreas development (Additional file 1: Table S1). Cells were coaxed to NKX6–1^+^/PDX1^+^ cells by employing previously published protocols [41, 42] and implanted in mice, resulting in functional β-cells and polyhormonal cells after 3–4 months.

This prompted an investigation into ways to promote further the in vitro maturation of hPSC-derived NKX6–1^+^/PDX1^+^ cells. As a result, cells were treated with factors including: betacellulin (a growth-promoting factor), triiodothyronine (T3, which is known to promote liver maturity although there is no evidence about its effect on pancreas development), XXI (a γ-secretase inhibitor for Notch signaling), a transforming growth factor-β (TGF-β) type I receptor kinase (ALK5) inhibitor (AXL) and N-acetyl cysteine promoting the production and nuclear localization of MAFA. From NKX6–1^+^/PDX1^+^ cells, β-cell-like cells (termed SC-β cells, ~33% NKX6–1^+^/C-peptide^+^) emerged in 2–3 weeks. Non-NKX6–1^+^/C-peptide^+^ cells in the final population were α-cells, δ-cells and PDX1^+^ pancreatic progenitors that had not differentiated into endocrine cells. SC-β cells responded to 2–3 successive glucose challenges in vitro by releasing insulin with a comparable stimulation index (ratio of insulin secretion at 20 mM to 2 mM glucose) to that of primary human adult islets, i.e. 2.2 ± 0.3 vs. 2.1 ± 0.9, respectively. Perifusion analysis was not reported but the SC-β cells secreted insulin in response to 20 mM glucose (1.6 ± 0.2 vs. 3.6 ± 0.7 μIU/10^3^ cells) and their total insulin content was similar to that of human adult islets (240 ± 50 μIU vs. 200 ± 40 μIU/10^3^ cells). Of note, 7.7 ± 0.7% and 4.7 ± 0.1% of C-peptide^+^ cells co-expressed GCG and SST, respectively.

More importantly, increased human insulin was observed in the blood stream of mice challenged with glucose after only 2 weeks post-implantation of the SC-β cells, most likely due to the additional maturation achieved with this protocol in vitro. This is a significantly shorter time frame than in previous studies [41, 42] and comparable to that seen in experiments involving the transplantation of human primary islets in rodents. Furthermore, implanted SC-β cells retrieved after 2 weeks were still monohormonal and transplantation of SC-β cells into a mouse model of hyperglycemia (NRG-Akita) restored normoglycemia for longer than 4 months.

In addition to the in vitro maturation, another important advancement was the differentiation of hPSCs (HUES8 hESCs and hiPSC-1 and hiPSC-2 hiPSCs) in aggregate cell cultures in 500-mL spinner flasks. While the yield of differentiated cells over the initial cell number was not reported and cells were not maintained under xeno-free conditions, the work illustrated the feasibility for the scalable production of β-cells [8]. Moreover, β-cells were produced with this protocol using hiPSCs from T1D patients, which demonstrated that these T1D SC-β cells function the same as normal SC-β cells [43].

Similar to the aforementioned report, Kieffer and colleagues devised a 7-stage protocol for driving the fate of hPSCs to pancreatic endocrine cells [9]. Cells differentiating to pancreatic endoderm progeny expressing PDX1/NKX6–1 (stages 1–4) were grown in a planar culture and then they were plated as clusters onto filter inserts (air-liquid interface (ALI) culture) for the remaining stages (Additional file 1: Table S1). In comparison to cells differentiated in two dimensional (2D) cultures, cells in ALI cultures exhibited upregulated *NGN3, INS* and *GCG* transcripts with almost 50% of cells being INS^+^ at stage 7. The majority of these cells co-expressed PDX1, NKX6–1 and MAFA. From a function standpoint, insulin release by stage 7 cells subjected to perifusion was delayed, gradual and low in comparison to human islets. Only a third of the cells responded to a glucagon-like peptide-1 (GLP-1) analog, displaying Ca^2+^ influx and an increase in intracellular Ca^2+^ after incubation with KCl. These data suggest that some of the differentiated cells have functioning incretin signaling pathways and voltage-gated Ca^2+^ channels. However, Ca^2+^ kinetics were slow in response to glucose exposure, pointing to deficiencies in glucose sensing/metabolism, insulin secretion machinery and/or the functionality of K^+^_ATP_ channels.

Problems in insulin secretion may be related to an insufficient quantity of easily releasable membrane-docked insulin vesicles or defective vesicle trafficking and exocytosis. The amount of membrane-docked insulin vesicles can be quantified by imaging, while experiments with K^+^_ATP_ channel blockers (e.g. tolbutamide) can be performed to address whether K^+^_ATP_ channels are functional. Unlike the generation of SC-β cells in stirred suspension cultures, it is unclear if ALI cultures are also scalable. Stage-7 cells also expressed MAFA at a significantly lower level compared to human islet cells. Nonetheless, the expression of MAFB was comparable between stage-7 cells and human islets. It is still debatable whether the expression of MAFA or MAFB is a representative metric of the state of hPSC-derived β-cell maturation. MAFA regulates β-cell maturation in rodents by regulating genes related to insulin synthesis, secretion and glucose sensing [44–46], while a recent study on human pancreatic cells has shown that MAFB (expressed both in α- and β-cells) remains unchanged with age [47].

It should be noted that hESC-derived glucose responsive β-cell-like cells in the aforementioned studies share many attributes with human adult β-cells [8, 9], but there are also considerable subpopulations of C-peptide^+^ cells expressing multiple hormones and lacking NKX6–1 expression. These cells most likely result from precocious endocrine differentiation of early PDX1^+^ cells which do not express NKX6–1 [48]. Such premature induction toward endocrine cell fates gives rise to polyhormonal cells and can be avoided by leaving out BMP inhibitors (e.g. noggin) during in vitro differentiation. Highly efficient generation of PDX1^+^/NKX6–1^+^ cells was demonstrated by using WNT3a/activin A (days 0–3) and KGF/TGF-β type I receptor kinase inhibitor IV (days 3–5), RA (days 5–7) and EGF/KGF (days 7–9) on hESC clusters (Additional file 1: Table S1), whereas the inclusion of noggin led to the premature emergence of NGN3^+^ cells. Further differentiation of these NGN3-expressing cells yielded endocrine cells which were co-positive for C-peptide and glucagon. In contrast, PDX1^+^/NKX6–1^+^ cells maintained in basal medium without additional growth factors proceeded to become C-peptide^+^ cells (23% at day 19), expressing β-cell transcription factors (PDX1, NKX6–1, NKX2–2, ISL1, PAX6, NEUROD1) and chromogranin A with only 3.2% of all differentiated cells being GCG^+^. The C-peptide^+^ cells had minimal proliferative capacity, expressed MAFA, MAFB and genes relevant to glucose metabolism (GCK), insulin processing (PC1/3) and membrane polarization (KIR6.2, SUR1). Transplantation of PDX1^+^/NKX6-1^+^ cells into STZ-treated diabetic mice resulted in significant reduction of blood glucose levels.

Most reports on pancreatogenic differentiation of hPSCs rely on trial-and-error experiments and the knowledge drawn from embryonic development of model organisms to select factors for coaxing hPSCs along pancreatic endoderm and islet cell fates. Recently, clustered regularly interspaced short palindromic repeats/Cas9 (CRISPR/Cas9) technology was utilized to knockout hPSC genes in a doxycycline-inducible manner [49] in an effort to dissect the action of specific genes during specification to pancreatic islet cells. *PDX1*, *RFX6* and *NGN3* knockouts had fewer INS^+^ or GCG^+^ cells as determined by immunocytochemistry and flow cytometry analysis, indicating that these genes are necessary for pancreatic endocrine differentiation. In contrast, knocking out *HES1* led to an increase of endocrine cells suggesting that HES1 expression inhibits pancreatic endocrine differentiation. In *ARX* mutants, the cells were not GCG^+^ (assessed by quantitative polymerase chain reaction (qPCR) analysis and immunostaining) and there was a 30% decrease in the number of INS^+^ cells as determined by flow cytometry**, which means** ***ARX*** aids in specifying GCG- and INS-expressing cells. At the pancreatic progenitor stage, mutants of *PTF1A*, *GLIS3*, *MNX1*, *NGN3*, *ARX* and *HES1* formed PDX1^+^ cells normally, while the RFX6 knockouts had a 40% decrease of PDX1^+^ cells suggesting that RFX6 is a regulator of PDX1 expression [49].

### Derivation of α-like cells

In addition to the production of insulin-secreting β-cells, reports are available on the derivation of other endocrine cell types, mainly α-cells, from hPSCs. Supraphysiological α-cell function and hyperglucagonemia are contributors to hyperglycemia in diabetic patients, stimulating glucose production in the liver [50]. The release of glucagon by α-cells is partly regulated by neighboring β-cell insulin secretion, which becomes aberrant in T2D. Thus, the in vitro generation of α-cells can serve as a resource for studies on glucagon-producing pancreatic cells in normal and diabetic states. Recent reports have also shown that α-cells can be converted into β-cells in mice following ablation of the existing β-cells [51] or ectopic expression of *pax4* [52], suggesting that stem cell-derived α-cells may act as a starting point for the generation of functional β-cells.

Human ESCs have been coaxed into α-cells using a 4-week, 6-stage regimen (Additional file 1: Table S1) [53]. Initially, cells were induced to an anterior primitive streak cell fate, followed by endocrine precursors and pancreatic endocrine phenotypes with cells forming spontaneously clusters that contained α-cells. The final population featured 99.3% SYP^+^ and 65.3% GCG^+^/INS^+^ cells as determined by flow cytometry vs. 61% and 4% found in human islets, respectively. Approximately 65% GCG^+^ cells (4.3% INS^+^/GCG^+^) were produced with extended culture of the clusters while the levels of SYP^+^ cells remained around 75%. Cells also stained positive for the transcription factor ARX, which contributes to α-cell development [54]. Also, the cells exhibited a 10-fold higher glucagon content vs. human islets. Conversely, the insulin content was 10 times less than in human islets and the cells exhibited very low insulin secretion in vitro in response to KCl and arginine, but not to glucose. Basal secretion of glucagon at low glucose was variable but on average ~ 4-fold higher than that of native islets. Incubation with arginine, KCl and carbachol (an acetylcholine analog) triggered the release of glucagon whereas octreotide (an octapeptide analog of somatostatin) and high glucose had the opposite effect, in line with their physiological roles.

The hESC-derived α-cells were transplanted to normoglycemic mice to assess glucagon secretion in vivo. Animals receiving the cells (Tx group) exhibited a significant increase in glucagon after intraperitoneal administration of arginine. When the mice were subjected to an oral glucose challenge, approximately 2.5 months after implantation, there were no differences in insulin secretion and glucose clearance between the control and Tx groups. Yet, the Tx group displayed chronic hyperglucagonemia and mild glucagon resistance. Removal and examination of the engrafted cells 4 months post-transplantation revealed that the cells maintained the expression of glucagon and other α-cell markers (ARX) but there was downregulated or undetectable expression of β-cell genes, such as *INS*, *MAFA* and *NKX6–1*. Additionally, the α-cell clusters did not revert to a different lineage fate upon implantation in STZ-treated mice, suggesting a robust in vitro commitment.

While more than half of the differentiated cells displayed α-cell markers, we surmise that these cells are not mature given that, upon transplantation in mice, GLP-1 plasma levels were increased in response to arginine administration. The presence of GLP-1 is noteworthy because it has previously been shown that prohormone convertase 1/3 (PC1/3), which converts proglucagon to GLP-1 and GLP-2 instead of glucagon, is expressed in immature rather than mature α-cells [55]. In addition, the peak in vivo glucagon levels after implantation increased about 4-fold from day 9 to 62, suggesting that further in vivo maturation of these cells is required. Therefore, similar to the derivation of insulin-producing cells from hPSCs, the in vitro maturation of α-cells is a major challenge to be tackled.

Overall, vast improvements in the in vitro differentiation of hPSCs to pancreatic endocrine cell progeny have been made in recent years. For both β- and α-cells the main difficulty at present is their in vitro maturation into cells that exhibit physiologically relevant hormone release profiles. Recent findings however have brought us closer to achieving the goal of in vitro maturation of hPSC-derived cells to functional endocrine cells.

### hPSC differentiation to pancreatic exocrine cells

While the connection between diabetes and the functions of the endocrine pancreas is well-established, the role of the exocrine compartment in the context of the disease is still unclear. A considerable fraction of patients with diabetes mellitus exhibit pancreatic exocrine insufficiency including altered exocrine morphology and function and increased inflammation [56, 57]. Hence, engineered exocrine pancreatic tissue equivalents can serve as in vitro models to study the physiology of the exocrine compartment in diabetes and potentially to reconstitute the function of exocrine cells damaged by the disease.

Protocols for specifying hPSCs to pancreatic exocrine cells are underdeveloped and are characterized by lower differentiation efficiency than those for the generation of endocrine β-cells. Takizawa-Shirasawa et al. [58] demonstrated the conversion of hESCs in 15 days to pancreatic exocrine cells generating 12.2 ± 6.1% amylase^+^ and carboxypeptidase A^+^ cells (Additional file 1: Table S2).

Recently, the differentiation of hPSCs toward exocrine cell fates was demonstrated in organoid cultures [59]. Three-dimensional organotypic culture methods have been developed for the differentiation of stem cells and the formation of miniature organ-like structures (organoids), featuring ultrastructural and functional similarities to their native tissue counterparts. Various organoid models have been reported, including brain [60, 61], kidney [62, 63], intestine [64, 65] and liver organoids [66, 67]. Even primary human pancreatic cells embedded in Matrigel or rat tail collagen proliferate and form cysts displaying ductal cell markers and structures reminiscent of endocrine islets [68]. Notably, undifferentiated hPSCs also form structures containing cysts or lumens when seeded in Matrigel gel [69].

**Fig. 3:**
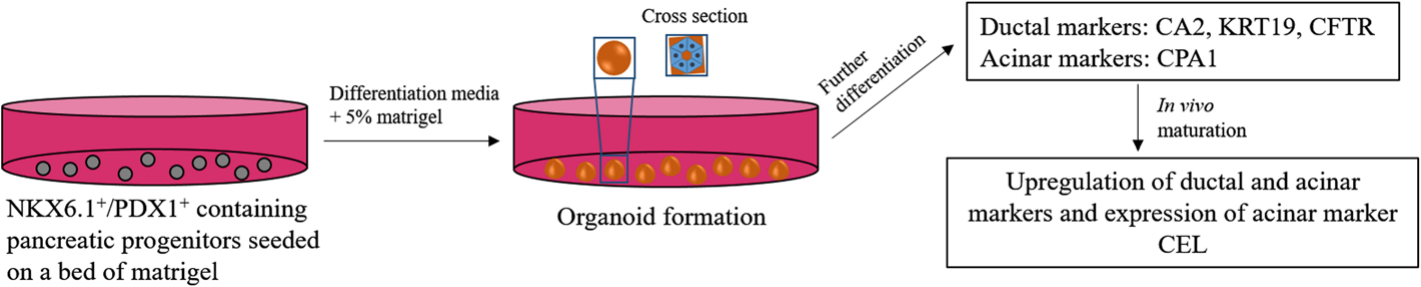
Schematic of the protocol for the creation of 3D organoid-like structures from NKX6.1^+^/PDX1^+^ containing pancreatic progenitors differentiated from hESCs

For exocrine differentiation in organoid cultures, hPSCs were first converted in monolayer cultures to NKX6–1^+^/PDX1^+^ progenitors before being placed on a Matrigel bed with differentiation media that contained 5% Matrigel for self-assembly into organoids with differentiation media that contained 5% Matrigel (Fig. 3, Additional file 1: Table S3) [59]. About 10–20% of the differentiated cells formed polarized organoids. The differentiation resulted in ductal and acinar cell populations. Organoids comprised one layer of polarized epithelia surrounding a hollow central lumen and most organoids were clonally derived. Progenitor organoids proliferated in culture for 12 days reaching diameters of 30–200 μm and day 16 organoids could be serially passaged. Cells were ~70% PDX1^+^, ~90% SOX9^+^ and ~77% NKX6–1^+^, while all the cells expressed the ductal marker KRT19. Starting on day 6, cells displayed low NGN3 expression and a 3.5-fold increase in SOX9 indicative of exocrine specification. However, ductal (CA2 and CFTR) and acinar (carboxyl ester lipase (CEL), pancreatic lipase (PNLIP) and serine peptidase inhibitor, Kazal type 1 (SPINK)) cell markers were either undetectable or at lower levels in organoids compared to adult pancreatic tissues, indicating that the organoid cells were mainly progenitor cells. These organoids gave rise to 10–15% CA2^+^ ductal and 0.5–1% CPA1^+^ acinar organoids. The reported fractions of exocrine cells in the organoids were very low in comparison to those achieved in the previously mentioned monolayer cultures. Further work is warranted for enhancing the efficiency of exocrine differentiation.

As a side note, the formation of organoids by cells from human pancreatic ductal adenocarcinoma (PDAC) biopsies was also demonstrated in the same report. Tumor organoids maintained some characteristics of the original PDAC making this system useful for studying pancreatic cancer and screening pertinent drugs for personalized therapeutic regimens [59, 70]. Furthermore, others have demonstrated the generation of acinar and ductal cells in organoids of hPSCs derived from cystic fibrosis patients as an in vitro model of the disease [71].

The efficiency of hPSC differentiation to exocrine cells is low compared to that toward endocrine cells. This is probably due to the emphasis placed predominantly on the derivation of insulin-producing cells. However, exocrine cells may be beneficial for in vitro models of diabetes, as mentioned previously. The organoid-like structures that contain some exocrine cells provide an interesting model [59], which recapitulates 3D architecture that is similar to in vivo pancreatic development. With further work, this model could potentially provide a way for more efficient generation of exocrine cells.

### Challenges and future directions

#### Protocol optimization and identification of factors for in vivo maturation of hPSC-derived pancreatic cell progeny

Despite significant recent advances in the differentiation of hPSCs into pancreatic cells, several challenges remain to obtain functional human pancreatic – particularly insulin-producing – cells performing on par with native cells. The variability noted in different reports in the specification outcomes across various hPSC lines (Table 1) points to the need for protocol adaptation and optimization, generally hindering direct translation to multiple lines. In addition, glucose-stimulated insulin secretion (GSIS) of many stem cell-derived pancreatic cells differs significantly from that of primary human islets. Furthermore, evidence of hPSC-derived islet performance under sequential glucose challenges is limited and the kinetics of GSIS are typically slow. More importantly, the underlying causes of these discrepancies between native islet cells and hPSC-derived cells are unclear. For instance, differences in glucose metabolism and deficiencies in hormone exocytosis and membrane docking of insulin granules have been suggested as potential culprits [9]. Yet, pancreatic progenitors from cultured hPSCs become glucose-responsive after implantation in mice illustrating the need for additional factors to drive the maturation of these cells in vitro.

In addition, no factors which promote the in vivo maturation of the hESC-derived pancreatic progeny have been identified [35]. This issue was explored by grafting hESC-derived pancreatic progenitor cells in immunodeficient rats and mice [72]. Although human C-peptide levels were higher in mice than rats, only rats had glucose-responsive human C-peptide secretion by weeks 19–21 and the cell grafts in rats had higher levels of mature β-cell genes with lower levels of *NGN3*, *GCG* and *ARX* than cells in mouse grafts. Compared to mouse grafts, rat grafts had a higher density and distribution of red blood cells and rats had lower levels of circulating human vascular endothelial growth factor C (VEGF-C) and basic FGF (bFGF). Thus, maturation of the transplanted cells may be facilitated by the inclusion of red blood cells and prevented by the presence of VEGF-C and bFGF. Rat grafts were also supported by a compact collagen network, which was looser in mice. The denser collagen network may promote cell maturation, which is in agreement with the findings that collagen IV-modified poly (lactide-co-glycolide) (PLG) scaffolds improve the GSIS of isolated mouse islets in comparison to serum-modified PLG scaffolds [73].

#### Large-scale culture systems

While work on the latter stages (maturation) of the hPSC differentiation to insulin-producing β-cells is ongoing, there is a glaring need for scalable bioprocesses for the generation of clinical grade β-cells for diabetes therapies. It is estimated that a dose of ~10^9^ β-cells is necessary for envisioned cell therapy protocols [4]. Whether the cells will be produced in a scale-out (multiple small batches each for a single patient) or scale-up (a large batch servicing multiple patients) fashion is debatable. Nonetheless, given that monolayer methods have inherent shortcomings for growing large amounts of cells, three dimensional (3D) systems are an appealing option for the expansion of hPSCs and their specification to pancreatic cells [8, 74].

Pancreatogenic differentiation has been demonstrated in shaker and spinner flasks, but these modalities lack automation for monitoring and controlling culture conditions. For example, while hPSCs are subjected to differentiation as aggregates in spinner flasks or rotary cultures over a few weeks (e.g. toward insulin-producing cells [8, 75]), cluster size increases due to cell proliferation, which is slower than self-renewing of hPSCs but nonetheless significant. As a result, mass transfer limitations, cell necrosis and aberrant differentiation can be exacerbated, especially when process monitoring and control are lacking. This hinders the downstream separation of the desired cell population(s). Frequent dissociation of the clusters is laborious, increases the risk for contamination, may decrease cell viability and has unexplored ramifications for cell specification and function.

Spinner flasks represent simplified versions of fully automated stirred suspension bioreactors, which are the workhorse of the biopharmaceutical industry for cell cultivation. Bioreactor systems, allowing the continuous monitoring and control of critical parameters such as pH, dissolved oxygen (DO), concentration of nutrients and metabolites, and feeding, can ensure the reproducibility and control of the expansion and directed differentiation. However, the dynamic bioreactor milieu is markedly different from the environment in static cultures and its effect(s) on the pancreatic specification of hPSCs has yet to be determined. To that end, application of the quality-by-design (QbD) framework that is routinely employed in pharmaceutical development [76, 77], can expedite the development of bioreactor processes for the production of stem cell therapeutics.

#### Large-scale postprocessing: Cell sorting

The production of medically relevant quantities of cells secreting insulin in response to glucose also hinges on technologies for the efficient and rapid separation of the desired cells from the usually heterogeneous populations of differentiated hPSCs. Fluorescence activated cell sorting (FACS) is customarily used in a laboratory setting for cell separation based on the expression of specific surface antigens [78–80] or fluorescent reporters [81] (after genetic manipulation of the cells). Alternatively, magnetic-activated cell sorting (MACS) can be used to obtain enriched cell populations. However, sorting rates typically do not exceed 25 × 10^3^ events/s (translating, practically, to a lower cell/s rate) and the post-sorting cell viability is less than 100%. Given that only a fraction of the differentiated cell ensemble exhibits appropriate markers, the time needed for isolating ~10^9^ β-cell-like cells per patient would be impractically long. In addition, this process might reduce cell viability and impair function because conventional cell sorting requires dispersing the aggregates, employed in most differentiation protocols, into single cells. If the phenotypic composition of the clusters is known and does not pose problems for downstream applications, large particle cytometry, which has been successfully applied for sorting 35–100 × 10^3^ human islets/h [82], could be utilized to process hPSC-derived islet clusters.

Sorting methods are based on the presence (positive selection) or absence (negative selection) of specific cell markers, but this does not guarantee that the selected cells exhibit desirable metabolic or functional attributes. For example, hPSC populations undergoing differentiation to heart cells can be incubated with lactate, which is utilized selectively by the cardiomyocytes, causing the elimination of undesired cells [83]. To our knowledge there is currently no such metabolism-based strategy for β-cell enrichment of stem cells undergoing pancreatogenic specification. hPSC-derived β-cell-like cells should display mechanisms of glucose sensing, insulin synthesis and bi-phasic secretion [84] akin to those in native β-cells. Methods for in vitro and in vivo assessment of insulin (and C-peptide) release after treatment with secretagogues are well-developed [85], but their results are an average insulin secretion rate that does not distinguish among individual cells or clusters based on their secretory capacity. In principle, this heterogeneity in hPSC-derived insulin-producing cells is mirrored in native β-cells [86], but GSIS of the former is typically significantly lower than that of the latter. Therefore, the question remains of how the ranges of GSIS rates for the hPSC-derived cells compare to those for human islets. Knowing the answer would facilitate not only the optimization of the differentiation protocols but also the selection of cells with high insulin production to maximize the ratio of secreted hormone over the number of cells needed for diabetes correction. Thus, novel technologies are needed for the rapid discrimination and harvest of medically relevant quantities of individual hPSC-derived β-cell-like cells or clusters with GSIS comparable to human pancreatic islet cells.

#### Cell loss during differentiation

Despite the significant progress in differentiation protocols, there are still substantial cell losses throughout commitment, for example at the onset of differentiation, or due to apoptosis, shear (in stirred suspension), cluster dissociation during culture (e.g., for re-seeding), and accumulation of metabolites. Such losses adversely affect the overall economics of the bioprocess, especially considering the high cost of xeno-free media, supplements and possibly scaffolds. This problem may be alleviated with the inclusion of molecules that limit cell apoptosis and sustain viability and proliferation (e.g. Z-VAD-FMK [87, 88], brain-derived neurotrophic factor (BDNF) and neurotrophin-3 and −4 [89]).

The diminished proliferative capacity of cells as differentiation progresses further compounds the reduction in the yield of hPSC-derived pancreatic cells. A possible solution to this is the establishment of self-renewing endodermal progenitor (EP) lines. For example, Cheng et al. [90] reported that after 3–4 weeks of hESC differentiation to hepatic cells, a subpopulation (EP cells) persisted morphologically resembling the undifferentiated ESC colonies. These cells expressed SOX17 and HNF4A but not NANOG and POU5F1. A fraction of EP cells co-expressed CXCR4/CD117/FOXA1/SOX17 and propagated extensively (>10^16^ expansion was demonstrated over 24 passages) forming endodermal tissues without tumorigenicity. The authors presented the derivation of EP cells from another hESC line and two hiPSC lines in a serum-free protocol under hypoxia and in a shorter time (5 days of endoderm differentiation). However, it is still unclear if hPSCs are predisposed to an EP cell fate or if the appearance of this population can be solely attributed to idiosyncrasies of the culture protocol.

#### Culture economics, efficiency, and efficacy

Improving the differentiation efficiency requires the presentation of physiologically relevant cues to cells, not only at specific concentrations, but also at precise times. However, biological ligands (e.g., activin, Wnt3a, FGFs etc.) are expensive and have variable activity depending on lot and storage length and have a short half-life in culture, making them unsuitable for large-scale production of pancreatic cells from hPSCs. To that end, small molecules with similar bioactivity to recombinant cytokines, longer half-lives, and consistent activity (e.g., SANT1 [91], LDN193189 [92]) can be used for making defined and xeno-free media for stem cell bioprocesses. These small molecules are less expensive and more stable, reducing the cost of culture and making it more applicable for large-scale culture. As the examples suggest, some of these small molecules exist, but ideally more should be developed to replace all the biological ligands needed for differentiation.

Considering the advantages of small molecules over growth factors and cytokines as differentiation agents, substantial effort has been focused on the discovery of new molecules for converting hPSCs to pancreatic cells. Advances in high-throughput screening methodologies have made the discovery of new molecules as differentiation stimuli possible. For example, Chen et al. reported the screening of ~5000 chemical compounds leading to the identification of (−)-indolactam V as an agent augmenting 10 times (vs. controls) the expression of PDX1 in hESC-derived DE cells [93]. Similarly, kinase inhibitors were screened by qPCR for the upregulation of endocrine marker expression in differentiating hPSCs [53].

A significant aspect of the in vivo differentiation process is the 3D architecture of the forming tissues. To that end, pancreatic organoids mimic ultrastructural attributes of the native organ compartments [70]. Organoid culture capitalizes on the cells’ ability to self-organize into luminal structures in conjunction with paracrine signaling. Huang et al. demonstrated the cultivation of hPSC-derived organoids displaying tight junctions (ZO-1 expression), a polarized epithelial (MUC1 expression) layer, basement membrane secretion (human collagen IV and laminin-α5), and hollow central lumen and apical microvilli [59]. These findings are in line with those reported for organoids in another study describing a hollow central lumen, polarized cell epithelial (apical marker ZO1 and basal marker laminin-α5 staining) and microvilli [71]. Of note, the pancreatic acini feature microvilli and a bud-like structure surrounding a lumen [14], which is similar to the hollow lumen of the organoids bordered by cells. Whether hPSC organoid cultures can be employed for the efficient generation of tissue equivalents of the pancreas as shown for other organs (e.g. retina [94], intestine [95], kidney [96], brain [60]) remains an open question, especially given that the production of pancreatic endocrine organoids has yet to be reported.

#### Exosomes and miRNAs in pancreatic differentiation

Significant effort is also devoted to the discovery of factors beyond the traditional soluble cues for pancreatogenic differentiation. To this end, exosomes transferring information among cells in the form of proteins, DNA and RNA, including microRNA (miRNA), may participate in the process of hPSC fate specification. Probing the cargo of exosomes produced during hPSC differentiation to pancreatic cell progeny may reveal clues about pathways engaged in the transition of hPSCs toward islet cells and their maturation. It is expected that with ongoing refinements in exosome isolation, more miRNA and other moieties may be revealed as potentially useful in augmenting the differentiation of hPSCs to pancreatic progeny. MiRNAs, whether in exosomes or natively expressed in cells, may also influence differentiation. For instance, hPSCs overexpressing miRNA-186 and miRNA-375 display pancreatic cell-specific transcription factors and genes, such as *INS*, *NGN3*, *GLUT2*, *PAX4*, *PAX6*, *NKX6–1*, *PDX1* and *GCG* [97]. Morphologically the cells resemble islet cells, staining positive for insulin, NGN3 and zinc-containing granules (dithizone), and they secrete insulin in response to glucose. Hence, the efforts to convert hPSCs to pancreatic endocrine cells may benefit from strategies capitalizing on the increasing knowledge about the roles of exosomes and/or miRNAs on stem cell commitment.

It should be noted that the immunoprofile of the stem cell-derived pancreatic progeny should be scrutinized. This is essential when considering the use of these cells for patients with T1D, given the autoimmune nature of T1D, where direct implantation of islet cells generated from patient-specific iPSCs could be problematic. To that end, modulation of the immunoreactivity of stem cell-derived pancreatic cells may require their genetic manipulation, posing significant hurdles for their clinical use. However, there is a long history of developing biomaterials for islet encapsulation, offering protection from the host’s immune system [98], and various such designs have been used successfully in clinical trials involving islet transplantation [99, 100].

While several challenges and possible future directions were presented here, this is by no means an exhaustive list. Nonetheless, this discussion provides a perspective on where the the research is headed on the differentiation of hPSCs to functional pancreatic cells for diabetes therapies.

## Conclusions

The field of directing the fate of human stem cells to pancreatic cells and especially to insulin-secreting β-cells has come a long way in the last decade. Thus far, several laboratories have demonstrated the conversion of hPSCs to cells producing insulin, although there are still differences relative to human β-cells, most notably the immature phenotype of the hPSC-derived endocrine cells. As a result, a significant effort is placed on the maturation of the islet (and exocrine) cells from stem cells. Equally important however, is the effort to tackle issues surrounding the scalable generation and selection, identification of factors for in vivo maturation, culture economics, efficiency, and efficacy and exosomes and miRNAs in pancreatic differentiation. Furthermore, overcoming problems with functional evaluation of endocrine cells that can be used to manage diabetes and possibly other maladies, such as chronic pancreatitis and pancreatic cancer. Overall, there is still work to be done in creating fully functional pancreatic tissues from hPSCs in vitro, but given the current pace of progress, stem cell-based cellular therapeutics for diabetes may be available sooner than expected.

## Additional file

Additional file 1: Table S1. hPSCs endocrine differentiation protocols. **Table S2.** hPSCs exocrine differentiation protocols. **Table S3.** hPSCs pancreatic organoid culture. (DOCX 50 kb)
