# Supplementary material for: Human pluripotent stem cell differentiation to functional pancreatic cells for diabetes therapies: Innovations, challenges and future directions
Source: J Biol Eng. 2017 Jul 3;11:21. doi: 10.1186/s13036-017-0066-3 (PMC5494890; doi:10.1186/s13036-017-0066-3)
Supplement: Additional file 1: Table S1. — hPSCs endocrine differentiation protocols. Table S2. hPSCs exocrine differentiation protocols. Table S3. hPSCs pancreatic organoid culture. (DOCX 50 kb) [file 13036_2017_66_MOESM1_ESM.docx]

Supplementary Table S1. hPSCs endocrine differentiation protocols

Differentiation toward β-cells

D’Amour et al [35]

| Stage 1 | Stage 2 | Stage 3 | Stage 4 | Stage 5 |
| --- | --- | --- | --- | --- |
| Definitive Endoderm | Primitive gut tube | Posterior foregut | Pancreatic endoderm and endocrine precursor | Hormone expressing endocrine cell |
| Activin A 100 ng/mL | CYC .25 µM | CYC .25 µM | DAPT 1 µM | Ex4 50 ng/ml |
| Wnt 3a 25 ng/mL (D0-1) | FGF10 50 ng/mL | FGF10 50 ng/mL | Ex4 50 ng/ml | IGF1 50 ng/ml |
|  |  | RA 2 µM |  | HGF 50 ng/ml |
| RPMI + 0-.2% FBS | RPMI+2% FBS | DMEM+1% B27 | DMEM+1% B27 | CMRL*+1% B27 |
| 2-4 days | 2-4 days | 2-4 days | 2-3 days | 3+ days |
| Monolayer differentiation on mouse embryonic feeder layer | | | | |

*CMRL media from Invitrogen: https://www.thermofisher.com/order/catalog/product/11530037

Pagliuca et al [8]

| Stage 1 | Stage 2 | Stage 3 | Stage 4 | Stage 5 | Stage 6 |
| --- | --- | --- | --- | --- | --- |
| Definitive Endoderm | Primitive gut tube | early pancreatic progenitors | pancreatic progenitors PDX1+/NKX6-1+ | Endoderm NKX6-1+/C-peptide+ | Stem-cell-derived β cells |
| Activin A 100 ng/mL | KGF 50 ng/mL | KGF 50 ng/mL | | Alk5i II 10 µM | Alk5i II 10 µM |
|  |  | SANT-1 0.25 µM | | SANT-1 0.25 µM |  |
| CHIR 3µM (D0-1) |  | RA 2 µM | | RA .1 µM | T3 1 µM |
|  |  | LDN .2 µM (only D7) | | T3 1 µM |  |
|  |  | PdBU.5 µM (only D7-9) | | XXI 1 µM |  |
|  |  |  |  | Betacellulin 20 ng/ml |  |
| S1 | S2 | S3 | | S5 | S6 |
| D0-4 | D4-7 | D7-14 | | D14-21 | D21-35 |
| Cell clusters in spinner flasks | | | | | |

S1 media: MCDB131 + 8mM D-(+)-Glucose+ 2.46 g/L NaHCO_3_ + 2% FAF-BSA + ITS-X 1:50.000 + 2mM Glutamax + 0.25mM Vitamin C + 1% P/S.

S2 media: MCDB131 + 8mM D-Glucose + 1.23 g/L NaHCO_3_ + 2% FAF-BSA + ITS-X 1:50.000 + 2mM Glutamax + 0.25mM Vitamin C + 1% P/S.

S3 media: MCDB131 + 8mM D-Glucose + 1.23 g/L NaHCO_3_ + 2% FAF-BSA + ITS-X 1:200 + 2mM Glutamax + 0.25mM Vitamin C + 1% P/S.

S5 media: MCDB131 + 20mM D-Glucose + 1.754 g/L NaHCO_3_ + 2% FAF-BSA + ITS-X 1:200 + 2mM Glutamax + 0.25mM Vitamin C + 1% P/S + Heparin 10μg/ml.

S6 media: CMRL 1066 Supplemented + 10% FBS + 1% P/S.

Rezania et al 2014 [9]

| Stage 1 | Stage 2 | Stage 3 | Stage 4 | Stage 5 | Stage 6 | Stage 7 |
| --- | --- | --- | --- | --- | --- | --- |
| Definitive Endoderm | Primitive gut tube | Posterior foregut | Pancreatic endoderm | Pancreatic endocrine precursors | Immature β cells | Maturing β cells |
| GDF8 100 ng/mL | KGF 50 ng/mL | KGF 50 ng/mL | KGF 50 ng/mL | ZnSO_4_ 10 μM | ZnSO_4_ 10 μM | ZnSO_4_ 10 μM |
| 1, .1, 0 μM (D0-3) MCX-928*  or CHIR | Ascorbic Acid 0.25 mM | SANT-1 0.25 µM | SANT-1 0.25 µM | SANT-1 0.25 µM | XXI .1 µM  (D13-20 only) | Trolox 10 μM |
|  |  | RA 1 µM | RA .1 µM | RA 0.05 μM |  | *N*-Cys 1 mM |
|  |  | LDN .1 µM | LDN .2 µM | LDN .1 µM | LDN .1 µM | R428 2 μM |
|  |  | TPB .2 µM | TPB .1 µM | Alk5i II 10 μM | Alk5i II 10 μM | Alk5i II 10 μM |
|  |  |  |  | T3 1 μM | T3 1 μM | T3 1 μM |
|  |  |  |  | Heparin 10 μg/ml | Heparin 10 μg/ml | Heparin 10 μg/ml |
| MCDB 131 | MCDB 131 | BLAR | BLAR | BLAR | BLAR | BLAR |
| D0-3 | D3-5 | D5-7 | D7-10 | D10-13 | For 7-15 days | For 7-15 days |
| Planar Culture (matrigel coated plates) | | | | Air-liquid interface | | |

*For iPSC line, 1.5 μM MCX-928 D1

MCDB 131 medium: with sodium bicarbonate (1.5 g/l), 1× Glutamax, 10 mM final glucose concentration, 0.5% BSA

BLAR medium: with 2.5 g/l for S3-4 and 1.5 g/l for S5-7 sodium bicarbonate. 1× Glutamax, 10 mM (S3-4) and 20 mM (S5-7) final glucose concentration, 2% BSA, 1:200 ITS-X. MCDB 131 could be substituted for BLAR media.

Zhu et al [49]

| Stage 1 | Stage 2 | Stage 3 | Stage 4 | Stage 5 | Stage 6 | Stage 7 |
| --- | --- | --- | --- | --- | --- | --- |
| Definitive Endoderm | Early Pancreatic Progenitor | | PDX1+NKX6.1+ progenitor cells | β cells like | | |
| GDF8 100 ng/mL | KGF 50 ng/mL | KGF 50 ng/mL | KGF 2 ng/mL | ZnSO_4_ 10 μM | ZnSO_4_ 10 μM | ZnSO_4_ 10 μM |
| CHIR  3 μM D1  .3 μM D2  0 μM D3 | Ascorbic Acid 0.25 mM | ascorbic acid 0.25 mM | ascorbic acid 0.25 mM | SANT-1 0.25 µM | XXI .1 µM | Trolox 10 μM |
|  |  | SANT-1 0.25 µM | SANT-1 0.25 µM | Heparin 10 μg/ml | Heparin 10 μg/ml | Heparin 10 μg/ml |
|  |  | RA 1 µM | RA .1 µM | RA 0.05 μM |  | *N*-Cys 1 mM |
|  |  | LDN .1 µM | LDN .2 µM | LDN .1 µM | LDN .1 µM | R428 2 μM |
|  | IWP-2 1.25 μM | TPB .2 µM | TPB .1 µM | Alk5i II 10 μM | Alk5i II 10 μM | Alk5i II 10 μM |
|  |  |  | IWP-2 1.25 μM | T3 1 μM | T3 1 μM | T3 1 μM |
| S1 | S1 | S3 | S3 | S5 | S5 | S5 |
| D0-3 | D3-4 | D5-6 | D7-9 | D10-12 | D13-19 | D20-33 |
| Planar Culture (matrigel coated plates) | | | | Air-liquid interface | | |

S1: MCDB 131, sodium bicarbonate (1.5 g/l), 1× Glutamax, 10 mM final glucose concentration, 0.5% BSA

S3: MCDB 131, sodium bicarbonate (2.5 g/l), 1× Glutamax, 10 mM final glucose concentration, 2% BSA, ITS-X 1:200

S5: BLAR, sodium bicarbonate (1.5 g/l), 1× Glutamax, 20 mM final glucose concentration, 2% BSA, ITS-X 1:200

Differentiation toward α-cells

Rezania et al 2011 [53]

| Stage 1 | Stage 2 | Stage 3 | Stage 4 | Stage 5 | Stage 6 |
| --- | --- | --- | --- | --- | --- |
| Mesoendoderm | Endoderm Progenitor | Foregut Progenitor | Endocrine Precursor | Immature Endocrine | Maturing Endocrine |
| Activin A 100 ng/mL | KGF 50 ng./mL | KGF 50 ng/mL | Alk5i II 1 µM | Alk5i II 1 µM | Alk5i II 10 µM |
| Wnt3a 20 ng/mL (D0-1) | CYC 0.25 μM | CYC 0.25 μM | Noggin 100 ng/mL |  |  |
| bFGF 8 ng/mL |  | Noggin 100 ng/mL | DAPT 1 µM |  | T3 1 µM |
|  |  | RA 2 µM |  |  |  |
| RPMI+2% BSA | DMEM/F12+2% BSA | DMEM/F12+1% B27 | DMEM/F12+1% B27 | DMEM/F12+1% B27 | DMEM/F12+1% B27 |
| D0-3 | D3-5 | D5-9 | D9-12 | D12-19 | D19-26 |
| Monolayer differentiation on Matrigel coated plates | | | | | |

Supplementary Table S2. hPSCs exocrine differentiation protocols

Takizawa-Shirasawa et al [58]

| Stage 1 | | Stage 2 | Stage 3 |
| --- | --- | --- | --- |
| Definitive Endoderm | | Pancreatic progenitor | Exocrine cells |
| Activin A 100 ng/ml | Activin A 100 ng/ml | 1 μM RA | FGF7 15 ng/ml |
| Wnt3a 25 ng/ml | Insulin 5 μg/ml |  | 10 mM Nicotinamide |
|  | Transferrin 50 μg/ml |  | GLP-1 100 ng/ml |
|  | Selenium Chloride 30nM |  |  |
| RPMI | RPMI | RPMI+2% FBS | DMEM/F12+N2+B27 |
| 1 day | 2 days | 3 days | Up to 15 days |

Supplementary Table S3. hPSCs pancreatic organoid culture

Huang et al [59]

| Stage 1 | | Stage 2 | Stage 3 | Stage 4 |
| --- | --- | --- | --- | --- |
| Definitive endoderm | | Foregut/Midgut | Pancreatic Endoderm | PDX-1+/NKX6.1+ progeni­tors |
| Activin A 100 ng/ml | Activin A 100 ng/ml | CYC .25 µM | CYC .25 µM | EGF 50 ng/ml |
| CHIR 1 μM | bFGF 2.5 ng/ml |  | Noggin 50 ng/ml | Noggin 50 ng/ml |
|  | Ascorbic Acid 0.5 mM |  | RA 2 μM | Nicotinamide 1.2 μg/ml |
|  |  |  | Ex-4 50 ng/ml | Ex-4 50 ng/ml |
| RPMI | RPMI | RPMI+1%B27 | DMEM | DMEM |
| D0-1 | D1-3 | D3-5 | D5-7 | D7-9 |
| Monolayer differentiation on mouse embryonic feeder layer | | | | |

| Stage 1 | Stage 2 | Stage 3 |
| --- | --- | --- |
| Organoid Culture | | |
| Ascorbic acid 50 µg/ml | 2-phosphoascorbic acid 300 μM | 2-phosphoascorbic acid 300 μM |
| Insulin 20 μg/ml | FGF7 100 ng/ml | FGF7 100 ng/ml |
| Hydrocortisone .25 μg/ml | EGF 10 ng/ml | EGF 10 ng/ml |
| bFGF 100 ng/ml | A8301 1 μM |  |
| RA 100 nM | DBZ 1 μM |  |
| Y267632 10 μM |  |  |
| DMEM+ 1% B27 | DMEM+ 1% B27 | DMEM+ 1% B27 |
| D9-11 | D11-17 | D17-21 |
| Plated on a bed of matrigel | | |

Pancreatic organoid maintenance medium: DMEM, 1% B27 and 50 μg/ml ascorbic acid, 5% Matrigel

**Differentiation Table Abbreviations**

| bFGF | basic Fibroblast growth factor aka FGF2 |
| --- | --- |
| CHIR | CHIR99021 |
| CYC | KAAD-cyclopamine |
| DAPT | N-[(3,5-Difluorophenyl)acetyl]-L-alanyl-2-phenyl]glycine-1,1-dimethylethyl ester |
| EGF | Epidermal growth factor |
| Ex4 | Exendin-4 |
| FAF-BSA | Fatty acid Free Bovine Serum Albumin |
| FBS | Fetal Bovine Serum |
| FGF10 | Fibroblast growth factor 10 |
| GDF8 | Myostatin |
| GLP-1 | Glucagon-like peptide-1 |
| HGF | Hepatocyte growth factor |
| IGF1 | Insulin-Like Growth Factor-1 |
| ITS-X | Insulin-Transferrin-Selenium-Ethanolamine |
| KGF | Keratinocyte growth factor aka FGF7 |
| LDN | LDN193189 |
| NaHCO_3_ | Sodium bicarbonate |
| N-Cys | N-acetyl cysteine |
| P/S | Penicillin-Streptomycin |
| PdbU | Phorbol 12,13-dibutyrate |
| RA | retinoic acid |
| T3 | Triiodothyronine |
| TPB | ((2S,5S)-(*E*,*E*)-8-(5-(4-(trifluoromethyl)phenyl)-2,4-pentadienoylamino)benzolactam |
| XXI | C₂₇H₂₄F₂N₄O₃ |
| ZnSO_4_ | Zinc Sulfate |

**Role of differentiation factors**

| **Function** | **Factor** |
| --- | --- |
| Antioxidant | N-acetyl cysteine (N-Cys) |
| AXL (tyrosine-protein kinase receptor) inhibition | R428 |
| Bone morphogenetic protein inhibition | Dorsomorphin, noggin, LDN193189 |
| EGF family member | betacellulin |
| Extracellular matrix production | Vitamin C |
| GLP-1 receptor agonist | Exendin-4 |
| Glycogen synthase kinase 3 (GSK3) inhibition | Wnt3a, CHIR99021 |
| Growth factors | KGF=FGF7, FGF10, bFGF=FGF2 |
| γ-secretase/notch inhibition | XXI, DAPT, DBZ |
| Hedgehog signaling inhibition | KAAD-cyclopamine (CYC), SANT-1 |
| Mitogen-activated protein kinase (MEK) inhibition | PD0325901 |
| Phosphatidylinositol 3-kinase (PI-3K) inhibition | Activin A |
| Protein kinase C activation | Phorbol 12,13-dibutyrate (PdbU), TPB, (−)-Indolactam V |
| Rock Inhibitor | Y267632 |
| TGF-β type I receptor inhibition | Alk5i, SB431542, A8301 |
| TGF-β family member | GDF8 aka Myostatin |
| Thyroid hormone | Triiodothyronine (T3) |
| Vitamins | Trolox (analog of vitamin E), Nicotinamide (vitamin B group), Vitamin C |
| Wnt inhibition | IWP-2 |
